# Comparative study on *Bartonella* infection in spleen and kidney of small mammals from Mile City and Lianghe County, Yunnan Province

**DOI:** 10.3389/fvets.2025.1554633

**Published:** 2025-04-16

**Authors:** Rong Fu, Jia-Xiang Yin, Ping He, Yan Chen, Yi Luo, Ping-Guo Liu, Shuang-Ling Guo

**Affiliations:** School of Public Health, Dali University, Dali, China

**Keywords:** *Bartonella*, small mammals, prevalence, phylogenetic analysis, Yunnan Province

## Abstract

**Background:**

Bartonellosis is a zoonotic infectious disease caused by *Bartonella* spp. Small mammals are the most important hosts of *Bartonella* and play an important role in its long-term maintenance and spread. The multi-organ studies help understand the *Bartonella* prevalence of hosts more systematically and comprehensively. This study aimed to investigate the prevalence of *Bartonella* in small mammals and explore the genetic diversity of the infected strains and the influencing factors from Mile City and Lianghe County, Yunnan Province.

**Methods:**

Small mammals were captured in Mile City and Lianghe County of Yunnan Province from July to August 2019. Spleen and kidney tissues were collected and the *gltA* gene was amplified to detect and analyze the prevalence of *Bartonella* in two regions and two organs.

**Results:**

The prevalence of *Bartonella* in small mammals was 14.29% (43/301). Lianghe County’s risk of infection was 3.79-fold (95%*CI*: 1.39–13.35) compared to that of Mile City. The risk of infection in *Rattus tanezumi* was increased by 90% compared to *Suncus murinus* (95%*CI*: 0.01–0.63). The small mammals with tail lengths > 132 mm infected by *Bartonella* were 6.34 folds than that with tail lengths ≤ 132 mm (95%*CI*: 1.87–23.39). The spleen had a higher infection rate of 12.11% (35/289) than the kidney at 7.33% (22/300) (*χ*^2^ = 4.966, *p* = 0.026). There were no statistically significant differences in the prevalence of *Bartonella* among small mammals with different habitats, sex, age, flea infestation status, body weight, body length, hindfoot length, and ear height. Five *Bartonella* species were isolated in seven species of small mammals*. Bartonella tribocorum* is the dominant species in both regions, and it has a genetic relationship with the zoonotic pathogen *Bartonella elizabethae*.

**Conclusion:**

This study showed the prevalence of *Bartonella* in small mammals from Mile City and Lianghe County of Yunnan Province was high, and there were more types of *Bartonella* infection species. The spleen was more conducive to the growth and reproduction of *Bartonella*. The results of the study will help to prevent and control *Bartonella* infection and transmission to humans from small mammals in the two regions and provide a reference basis for further research on *Bartonella* infection in Yunnan or other similar regions.

## 1 Introduction

*Bartonella* spp. is a group of nutritionally demanding, facultative intracellular parasitic gram-negative aerobic bacteria belonging to the class *Proteobacteria*, subclass *Alpha proteobacteria*, order *Rhizobiales*, family *Bartonellaceae*, genera *Bartonella*. *Bartonella* is transmitted by blood-sucking arthropods, since its first isolation, new strains and species have been discovered, and more than 40 species and subspecies of *Bartonella* have been identified, 17 of which are associated with human infection (1, 2). With the continuous discovery of *Bartonella* species, the host animals suitable for storing *Bartonella* have increased exponentially, and due to the high heritability and diversity of *Bartonella* in rodents, rodents are the most important hosts of *Bartonella* and play an important role in its long-term maintenance and spread (1, 3). Previous studies have shown that the prevalence of *Bartonella* in foreign rodents ranges from 4.9% to 85%, and the common infection strains are *Bartonella grahamii* and *Bartonella phoceensis* (4–6). Rodents commonly associated with *Bartonella* infection in China are *Rattus* and *Apodemus*, with prevalence as low as 6.4% and as high as 57.7%, covering a wide range of areas, and the predominant strain of infection in many regions being *Bartonella grahamii*, which is a danger to human health, there is a need to investigate the prevalence of *Bartonella* in small mammals from different regions of the country (7–10). In addition, researchers performed multi-tissue *Bartonella* detection in rodents from northern and eastern China to obtain more accurate infection rates and more strain sequences for evaluation (9, 11).

Yunnan Province is located in the southwestern border mountainous area of China, the land area is large, the climate types are complex and diverse, and the special geographical environment and climate conditions provide an excellent growth environment for a variety of pathogenic microorganisms, increasing the risk of natural focal disease (8, 12). According to previous research findings, studies on *Bartonella* species prevalence in small mammals conducted in Heqing County and Gongshan County of Yunnan Province have primarily focused on assessing the genetic diversity and tissue tropism of *Bartonella*, while lacking cross-regional comparative analyses of regional differences (13). The study conducted in Yulong, Jianchuan, and Lianghe counties of Yunnan Province focused on comparing multifactorial differences across regions but did not involve multi-tissue testing (8). In light of the current research status on *Bartonella* infection in small mammals in Yunnan Province, it is imperative to integrate multi-tissue detection with cross-regional investigation of infection-influencing factors, building upon the key focus areas identified in previous studies. This comprehensive approach will contribute to a more accurate representation of *Bartonella* infection prevalence and its determinant factors among small mammals across different regions of Yunnan Province. The present study was based on two types of tissues for pathogen detection, aiming to investigate the infection status of *Bartonella* in small mammals from Mile City in eastern Yunnan Province and Lianghe County in western, to compare and analyze the epidemiological distribution of *Bartonella* in different regions, and to explore the influencing factors of the occurrence of *Bartonella* infections, to provide scientific basis for effective prevention and control of *Bartonella* in the region.

## 2 Materials and methods

### 2.1 Ethics statement

All methods were performed in accordance with the Medical Ethics Committee of Dali University. This Experimental Protocol has been reviewed and approved by the Medical Ethics Committee of Dali University (No. MECDU-201901-3) and conforms to the principles of medical experiment ethics and the relevant provisions of the national medical experiment ethics and welfare. Endangered or protected animal species were not included in this research.

### 2.2 Small mammal collection

**Figure 1:**
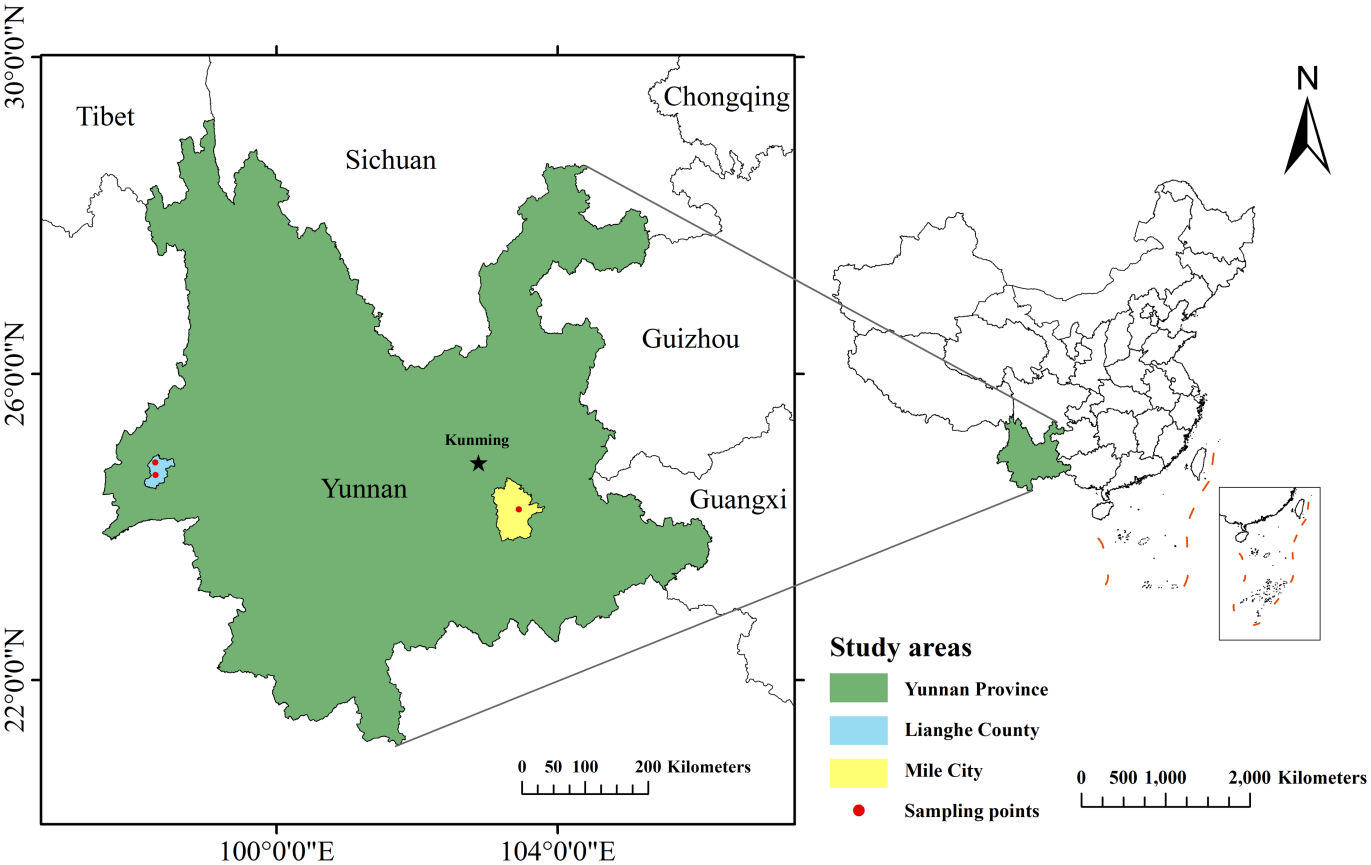
Map of areas sampled for small mammal capture.

Small mammals in Mile City and Lianghe County of Yunnan Province were captured using the night clamping method from July to August 2019, employing dead traps (15 × 8 cm) baited with peanuts. The areas for the trapping setting were selected and suggested by experts from the Center for Disease Control and Prevention of Mile and Lianghe in different landscapes (Residential areas, Cultivation, Shrubland, and Woodland), they provided the setting where small mammals captured were the most based on annual small mammals monitoring data. The sampling area map was constructed by ArcGIS 10.8, the sampling points are specified down to the township level, as shown in Figure 1. Within the selected area, place dead traps at approximately 20-meter intervals based on the specific activity traces of small mammals, to ensure coverage of the target species’ activity range and minimize repeated captures. Each sampling point should have no fewer than 200 traps deployed daily. All dead traps were placed the whole night starting from the evening of the day to the next early morning. As small mammals captured were already deceased upon collection, no anesthesia or euthanasia was performed in this study. Then, the captured small mammals were recorded with the geographical landscape elements where they were located on notes and transported back to the laboratory in individual bags (“one mammal per bag”). Species of small mammals were identified by morphological methods, and the mammals were sequentially numbered while recording their characteristics. Repeated freeze–thaw cycles can rupture cellular structures, releasing more nucleases and leading to DNA degradation. Increased frequency of freeze–thaw cycles progressively compromises DNA quality, as repeated temperature fluctuations negatively impact the integrity of biomolecules in tissues. To minimize degradation, it is recommended to divide fresh tissue into small aliquots before freezing for optimal preservation of biological samples (14). In addition, ultra-low temperature storage can inhibit ice crystal growth through vitrification, reduce molecular motion, maintain tissue integrity, and prolong storage duration. Therefore, in this study, spleen and kidney tissues collected under aseptic conditions were divided into multiple tubes and preserved through low-temperature transportation to −80°C freezers for storage.

### 2.3 DNA extraction and PCR

Approximately 10 mg of spleen or kidney sample was taken, and DNA was extracted according to the manufacturer’s instruction of TIANamp Genomic DNA Kit (DP304; TIANGEN; Beijing; China). The concentration and purity of each DNA sample were determined by an ultraviolet–visible spectrophotometer. Qualified DNA samples have concentrations greater than 50 ng/μl (50 μg/mL), and the A_260_/A_280_ was between 1.8 to 2.1. DNA samples that met the criteria were stored at minus 80°C until subsequent molecular experiments, while those that did not meet the criteria were subjected to a secondary re-extraction.

The citrate synthase (*gltA*) gene sequence was amplified by polymerase chain reaction (PCR) with reference to the previous study (15). The primer sequences are BhCS.781p:5′-GGGGACCAGCTCATGGTGG-3′ and BhCS.1137n:5′-AATGCAAAAAGAACAGTAAACA-3′. The reaction mixture (25 μL) contains the following components: 1 μL (10 μmol/L) of each primer, DreamTaq Green PCR Master Mix (2X) 12.5 μL, 3 μL of DNA template, 7.5 μL double-distilled H_2_O. *GltA* amplification was performed under the following conditions: one cycle for 3 min at 94°C; 30 cycles for 30s at 94°C, 30s at 53°C, and 1 min at 72°C; and a final extension for 5 min at 72°C. Next, PCR products with 379 bp were identified by 1.5% agarose gel containing 4SGelred (Sangon Biotech (Shanghai) Co., Ltd.) and visualized under the Gel imaging system (G: BOX F3, Syngene, American). The samples were subsequently subjected to Sanger sequencing (dideoxy chain-termination method) performed by Sangon Biotech (Shanghai, China) Co., Ltd. The final sequencing was successfully determined to be positive, at the same time, the detection of *Bartonella* in one tissue of the small mammal was judged to be an infection.

### 2.4 Phylogenetic analysis

The successfully detected sequences were edited and trimmed by the SeqMan program in DNASTAR Lasergene (*7.1 version*). Reference sequences encoding *gltA* of *Bartonella* were retrieved from GenBank by using the Basic Local Alignment Search Tool at the National Center for Biotechnology Information website.^1^ Sample sequences were aligned with reference sequences using Sequence distance in MegAlign of DNASTAR Lasergene. Phylogenetic analysis was performed with Clustal W protocol (default parameters) by MEGA software (*11.0 version*). The phylogenetic tree was created by using the Neighbor-joining method, and bootstrap values were calculated with 1,000 replicates. The outgroup used was *Brucella melitensis* (*gltA* gene accession number: NZ_ACEM01000037). For *Bartonella* species, the following *gltA* gene sequences were included: *Bartonella tribocorum* (OR117609), *Bartonella queenslandensis* (MH748120), *Bartonella phoceensis* (AY515126), *Bartonella mastomydis* (OQ305211), and *Bartonella clarridgeiae* (MH019300). The phylogenetic tree was modified using iTOL v6^2^ to add comments on host and strain isolation.

### 2.5 Statistical analysis

Statistical analysis was performed using R software (4.4.0 version). The precise probability approach was used to compute the overall 95% confidence intervals for each sample rate. A total of 301 small mammals were captured. Depending on their constituent ratio, small mammals were classified as dominant (>10%) or other (≤10%). Among them, 288 small mammals had their spleen and kidney collected simultaneously. McNemar’s test was used to compare the difference in infection rate among different organs of the same small mammal. The Chi-squared test was used to compare the difference in *Bartonella* infection rate among 301 small mammals in different regions, habitats, species, sex, ages, flea-carrying status, and appearance characteristics. The appearance characteristics of small mammals showed skewness and were grouped by median. In the analysis of infection influencing factors, variables with statistical significance (*p* < 0.05) were initially screened using univariate analysis (Chi-square test). These selected variables were then incorporated into a multivariate logistic regression model. Subsequently, the stepwise bidirectional regression method was employed for variable selection and model optimization. Multi-factor Logistic regression analysis was performed to identify the influencing factors of *Bartonella* infection in small mammals. The test level was *α* = 0.05.

## 3 Results

### 3.1 Species of small mammals and *Bartonella* detection

In total, 301 small mammals were trapped and identified as belonging to 3 orders, 4 families, 7 genera, and 11 species, with a wide range of species, as shown in Table 1. Overall, *Rattus tanezumi* was the dominant species accounting for 48.17% (145/301), with a 22.07% (32/145) infection rate in *Bartonella*, followed by *Suncus murinus* with 26.25% (79/301) account and a 1.27% (1/79) infection rate. In Mile City, the dominant species was *Rattus tanezumi* 44.12% (30/68) with an infection rate of 3.33% (1/30), followed by the *Mus caroli* 17.65% (12/68) with an infection rate of 8.33% (1/12). The dominant species in Lianghe County, consistent with the overall situation, were *Rattus tanezumi* 49.36% (115/233) with an infection rate of 26.96% (31/115) and the *Suncus murinus* 31.76% (74/233) with an infection rate of 1.35% (1/74). The prevalence of *Bartonella* in small mammals is shown in Table 1.

**Table 1:** Prevalence of *Bartonella* in small mammals captured from Mile City and Lianghe County, Yunnan province [Positive/*N*(%)].

Orders	Families	Genera	Species	Mile City	Lianghe County	Total
*Eulipotyphla*	*Soricidae*	*Suncus*	*Suncus murinus*	0/5 (0)	1/74 (1.35)^*^	1/79 (1.27)^*^
		*Crocidura*	*Crocidura attenuata*	0/4 (0)	0/6 (0)	0/10 (0)
			*Crocidura indochinensis*	0	0/2 (0)	0/2 (0)
	*Erinaceidae*	*Hylomys*	*Hylomys suillus*	0	0/11 (0)	0/11 (0)
*Rodentia*	*Muridae*	*Niviventer*	*Niviventer confucianus*	0	3/4 (75.00)	3/4 (75.00)
		*Rattus*	*Rattus nitidus*	2/4 (50.00)	0/2 (0)	2/6 (33.33)
			*Rattus andamanensis*	0/3	1/7 (14.29)	1/10 (10.00)
			*Rattus tanezumi*	1/30 (3.33)^*^	31/115 (26.96)^*^	32/145 (22.07)^*^
		*Mus*	*Mus caroli*	1/12 (8.33)^*^	0	1/12 (8.33)
			*Mus pahari*	0/5 (0)	3/7 (42.86)	3/12 (25.00)
*Scandentia*	*Tupaiidae*	*Tupaia*	*Tupaia belangeri*	0/5 (0)	0/5 (0)	0/10 (0)
-	-	-	Total	4/68 (5.88)	39/233 (16.74)	43/301 (14.29)

*, Dominant species; Positive, Positive samples; N, Detection samples; %, Detection rate of positives.

### 3.2 Distribution of *Bartonella* in different tissues of small mammal

A total of 43 small mammals were judged to be infected with *Bartonella*, with an infection rate of 14.29% (43/301). There were 7 species of small mammals infected with *Bartonella*: *Rattus tanezumi*, *Suncus murinus*, *Mus pahari*, *Rattus andamanensis*, *Mus caroli*, *Rattus nitidus,* and *Niviventer confucianus*, as shown in Table 2.

**Table 2:** Positivity rate of *Bartonella* infection in different organs of small mammals.

Host	Spleen		Kidney		Total	
	Detection samples	Positive samples (%)	Detection samples	Positive samples (%)	Detection samples	Positive samples (%)
*Rattus tanezumi*	140	25 (17.86)	144	19 (13.19)	145	32 (22.07)
*Suncus murinus*	79	0 (0)	79	1 (1.27)	79	1 (1.27)
*Mus pahari*	12	3 (25.00)	12	0 (0)	12	3 (25.00)
*Hylomys suillus*	11	0 (0)	11	0 (0)	11	0 (0)
*Tupaia belangeri*	10	0 (0)	10	0 (0)	10	0 (0)
*Rattus andamanensis*	10	1 (10.00)	10	1 (10.00)	10	1 (10.00)
*Crocidura attenuata*	8	0 (0)	10	0 (0)	10	0 (0)
*Mus caroli*	8	1 (12.50)	12	0 (0)	12	1 (8.33)
*Rattus nitidus*	6	2 (33.33)	6	0 (0)	6	2 (33.33)
*Niviventer confucianus*	4	3 (75.00)	4	1 (25.00)	4	3 (75.00)
*Crocidura indochinensis*	1	0 (0)	2	0 (0)	2	0 (0)
Total	289	35 (12.11)	300	22 (7.33)	301	43 (14.29)

In this study, 289 spleen samples and 300 kidney samples were tested. The infection rate of the spleen was 12.11% (35/289) and the kidney was 7.33% (22/300), which was higher in the spleen than in the kidney (*χ*^2^ = 4.966, *p* = 0.026), as shown in Table 2.

### 3.3 Analysis of infection difference and influencing factors in different characteristics of small mammals

Among 301 small mammals, the infection rate in Lianghe County was 16.74% (39/233) higher than that in Mile City at 5.88% (4/68) (*χ*^2^ = 5.066, *p* = 0.024). The infection rate of small mammals was 13.56% (8/59) in residential areas, 9.09% (12/132) in cultivation, 23.08% (18/78) in Shrubland and 15.63% (5/32) in woodland, with statistically significant rates of *Bartonella* infestation in the different habitats (*χ*^2^ = 7.905, *p* = 0.048), and the habitats of scrubland have the highest infection rate. The prevalence was 22.07% (32/145) in the dominant species of *Rattus tanezumi*, 1.27% (1/79) in the *Suncus murinus*, and 12.99% (10/77) in the other non-dominant species of small mammals, with statistically significant differences (*χ*^2^ = 18.216, *p* < 0.001), and *Rattus tanezumi* had the highest prevalence in this study. In terms of the physical characteristics of the small mammals, the prevalence with body weight > 68 g was 21.71% (33/152) higher than that of those with body weight ≤ 68 g, which was 6.71% (10/149) (*χ*^2^ = 13.824, *p* < 0.001). The infection rate of small mammals with body length > 141 mm was 18.37% (27/147) higher than that of body length ≤ 141 mm 10.39% (16/154) (*χ*^2^ = 3.909, *p* = 0.048); the tail length > 132 mm was 23.81% (35/147) higher than that of tail length ≤ 132 mm infection rate 5.19% (8/154) (*χ*^2^ = 21.283, *p* < 0.001); hindfoot length > 26 mm infection rate was 23.29% (34/146) higher than hindfoot length ≤ 26 mm 5.81% (9/155) (*χ*^2^ = 18.763, *p* < 0.001) and the infection rate of ear height > 17 mm was 22.14% (31/140) higher than ≤17 mm 7.45% (12/161) (*χ*^2^ = 13.196, *p* < 0.001). In addition, there was no statistical difference in *Bartonella* infection rate among different sex, ages, and flea-carrying status in small mammals, as shown in Table 3.

**Table 3:** Univariate analysis of *Bartonella* infection rates in small mammals collected from Mile City and Lianghe County, Yunnan province.

Variables	Detection samples	Positive samples	Prevalence (95%*CI*, %)	*χ* ^2^	*p*
Area				5.066	0.024
Mile	68	4	5.88 (1.63~14.38)		
Lianghe	233	39	16.74 (12.18~22.16)		
Landscape				7.905	0.048
Residential areas	59	8	13.56 (6.04~24.98)		
Cultivation	132	12	9.09 (4.79~15.34)		
Shrubland	78	18	23.08 (14.29~34.00)		
Woodland	32	5	15.63 (5.28~32.79)		
Species				18.216	<0.001
*Rattus tanezumi*	145	32	22.07 (15.61~29.70)		
*Suncus murinus*	79	1	1.27 (0.03~6.85)		
Others	77	10	12.99 (6.41~22.59)		
Sex				0.056	0.814
Female	159	22	13.84 (8.88~20.20)		
Male	142	21	14.79 (9.39~21.71)		
Age				5.82e^−30^	1.000
Immaturity	34	5	14.71 (4.95~31.06)		
Adult	267	38	14.23 (10.27~19.01)		
Flea infestation				0.04	0.842
No	272	38	13.97 (10.08~18.67)		
Yes	29	5	17.24 (5.85~35.77)		
Body weight				13.824	<0.001
≤68 g	149	10	6.71 (3.27~12.00)		
>68 g	152	33	21.71 (15.44~29.12)		
Body length				3.909	0.048
≤141 mm	154	16	10.39 (6.06~16.32)		
>141 mm	147	27	18.37 (12.47~25.59)		
Tail length				21.283	<0.001
≤132 mm	154	8	5.19 (2.27~9.98)		
>132 mm	147	35	23.81 (17.18~31.53)		
Hindfoot length				18.763	<0.001
≤26 mm	155	9	5.81 (2.69~10.74)		
>26 mm	146	34	23.29 (16.70~31.00)		
Ear height				13.196	<0.001
≤17 mm	161	12	7.45 (3.91~12.66)		
>17 mm	140	31	22.14 (15.57~29.93)		
Total	301	43	14.29 (10.54~18.76)		

The results of the single factor Chi-square test for different characteristics of small mammals showed that there were statistical differences in the prevalence of *Bartonella* in different areas, habitats, species, body weight, body length, tail length, hind foot length, and ear height. The stepwise regression method was used to incorporate these variables into the model, and finally, three variables (areas, species, and tail length) entered the model. The risk of *Bartonella* infection in Lianghe County was 3.79 folds higher than that in Mile City (95%*CI*: 1.39–13.35). *Rattus tanezumi* had a 90% (95%*CI*:0.01–0.63) increased risk of *Bartonella* infection compared to *Suncus murinus*. The risk of *Bartonella* infection in small mammals with tail length > 132 mm was 6.34 folds that of ≤132 mm (95%*CI*: 1.87–23.39). The analysis results are shown in Table 4.

**Table 4:** Multivariate logistic regression analysis for *Bartonella* prevalence in small mammals.

Variables	*b*	SE	OR (95%*CI*)	*p*
Area				
Mile	-	-	-	-
Lianghe	1.331	0.564	3.79 (1.39~13.35)	0.018
Species				
*Rattus tanezumi*	-	-	-	-
*Suncus murinus*	−2.296	1.111	0.10 (0.01~0.63)	0.039
Others	−0.026	0.452	0.97 (0.39~2.33)	0.954
Tail length				
≤132 mm	-	-	-	-
>132 mm	1.864	0.640	6.34 (1.87~23.39)	0.004

-, For the reference group; b, Coefficient; SE, Standard error; OR, Odds ratio.

### 3.4 Identifications of *Bartonella* species and distribution in small mammals

Fifty-seven strain sequences were obtained from 43 small mammals infected with *Bartonella*, of which 5 strains were isolated from *Rattus tanezumi*, *Rattus nitidus,* and *Mus caroli* in Mile City, 52 strains were isolated from *Rattus tanezumi*, *Suncus murinus*, *Niviventer confucianus*, *Rattus andamanensis* and *Mus pahari* in Lianghe County. Five species were identified through *gltA* gene analysis, including 32 strains of *Bartonella tribocorum*, the homology ranged from 91.5% to 99.3%; 11 strains of *Bartonella queenslandensis*, exhibited a similarity of 92.5% to 98%; 8 strains are closely related to *Bartonella phoceensis* (98.3%~99.8%); 4 strains of *Bartonella mastomydis* with identities between 95.5%~99.3%; 2 strains of *Bartonella clarridgeiae* were 93.3% and 95.8% homologous, respectively. The bacterial strains of small mammals in different regions are shown in Table 5.

**Table 5:** Distribution of *Bartonella* species among small mammals in Mile City and Lianghe County, Yunnan Province.

Species	Mile City			Lianghe County					Total
	*Rattus tanezumi*	*Rattus nitidus*	*Mus caroli*	*Rattus tanezumi*	*Suncus murinus*	*Niviventer confucianus*	*Rattus andamanensis*	*Mus pahari*	
*Bartonella tribocorum*	2	1	0	28	1	0	0	0	32
*Bartonella queenslandensis*	0	1	1	2	0	4	0	3	11
*Bartonella phoceensis*	0	0	0	6	0	0	2	0	8
*Bartonella mastomydis*	0	0	0	4	0	0	0	0	4
*Bartonella clarridgeiae*	0	0	0	2	0	0	0	0	2
Total	2	2	1	42	1	4	2	3	57

According to the differences in tissues, four *Bartonella* species were isolated from kidney tissues, including 3 strains of *Bartonella mastomydis*, 2 strains of *Bartonella phoceensis*, 1 strain of *Bartonella queenslandensis,* and 16 strains of *Bartonella tribocorum*. Five *Bartonella* species were isolated from the spleen: 2 strains of *Bartonella clarridgeiae*, 1 strain of *Bartonella mastomydis*, 6 strains of *Bartonella phoceensis*, 10 strains of *Bartonella queenslandensis* and 16 strains of *Bartonella tribocorum*. Among them, *Bartonella* strains were isolated from both tissues of 14 small mammals, and the *Bartonella* species isolated from different tissues of 13 small mammals were the same, but different species of strains were isolated from different tissues of *Rattus tanezumi* No. P016. The strain isolated from the kidneys of *Rattus tanezumi* P016 was *Bartonella mastomydis*, while that from the spleen was *Bartonella tribocorum*.

### 3.5 Phylogenetic tree construction

Fifty-seven samples were successfully sequenced, 5 reference strains based on *gltA* were selected in GenBank after BLAST to construct a phylogenetic tree, and *Brucella*, which was closely related to *Bartonella*, was selected as an outgroup. The phylogenetic tree of *gltA* showed that 57 strains were divided into five distinct clades, including 32 strains in the *Bartonella tribocorum* branch; 11 strains in the *Bartonella queenslandensis* branch; 8 strains in the *Bartonella phoceensis* branch; 4 strains in the *Bartonella mastomydis* branch; and 2 strains in the *Bartonella clarridgeiae* branch.

**Figure 2:**
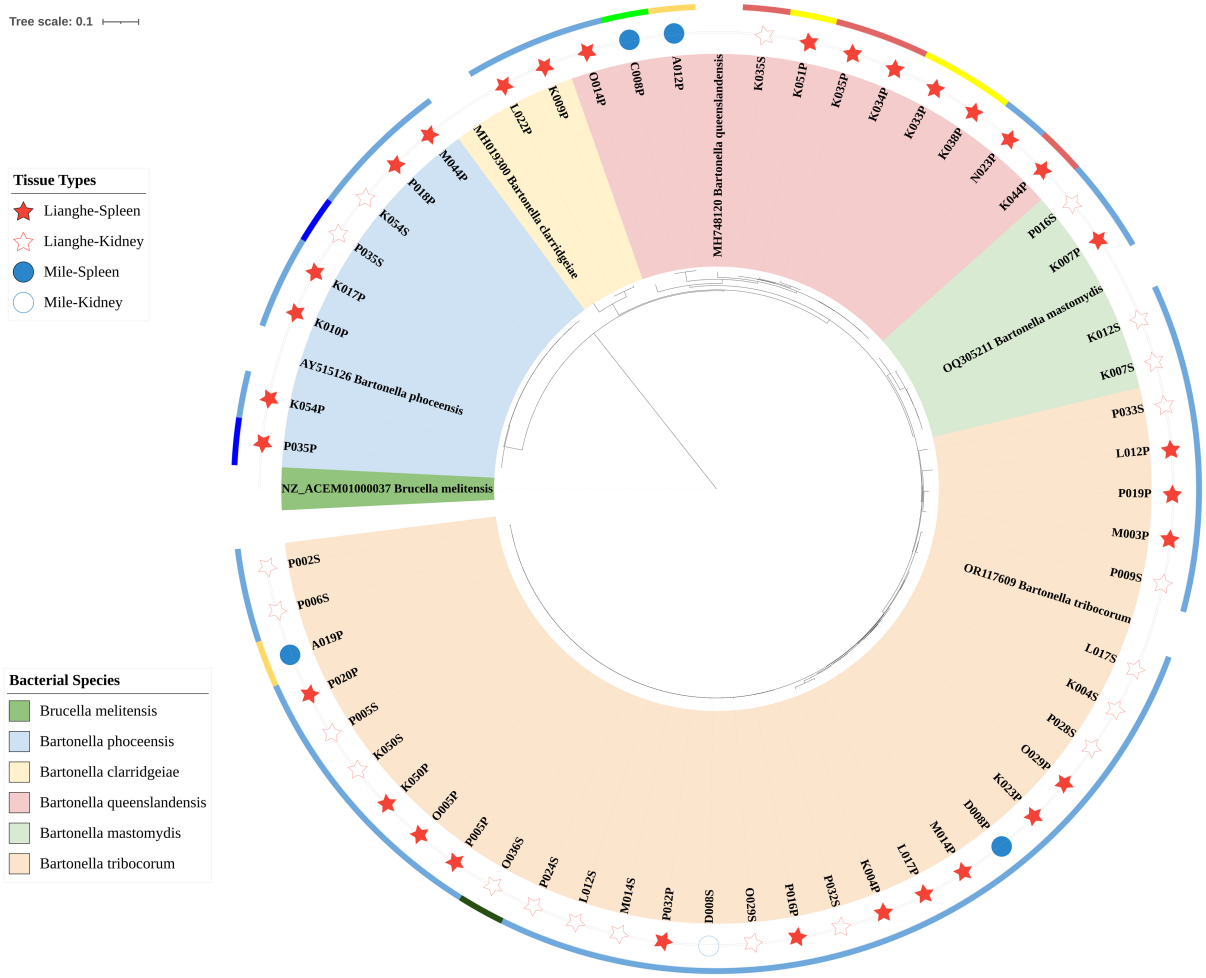
The phylogenetic tree was constructed based on the *gltA* gene.

The phylogenetic tree constructed based on the *gltA* gene is shown in Figure 2, where the red pentagram represents the Lianghe sample, the blue circle represents the Mile sample, the solid is the spleen sample, and the hollow is the kidney sample. The different colors of the outer ring represent the different small mammals: dark blue for the *Rattus andamanensis*, fluorescent green for the *Mus caroli*, dark green for the *Suncus murinus*, light blue for the *Rattus tanezumi*, light red for the *Niviventer confucianus*, orange for the *Rattus nitidus*, and yellow for the *Mus pahari*. Different branching colors represent different species of *Bartonella*.

## 4 Discussion

*Bartonella* is an emerging zoonotic pathogen that can be transmitted by blood-sucking arthropods. Since *Bartonella was* first isolated, it has been studied in various countries around the world, and rodents are important reservoir hosts for *Bartonella*, with the prevalence of *Bartonella* varying by region (2, 16). In recent years, the prevalence of *Bartonella* has been studied in many provinces in China, and the results are different (8, 10, 17–20). Therefore, it is important to investigate the prevalence of *Bartonella* with multiple organs among rodents in different areas.

A total of 301 small mammals were captured in this study and identified as belonging to 11 species, indicating the rich composition of small mammal species in Mile City and Lianghe County, Yunnan Province. The overall dominant rodent species in the two regions are the *Rattus tanezumi* and the *Suncus murinus*, and Lianghe County is consistent with the overall situation, but the dominant rodent species in Mile City are the *Rattus tanezumi* and the *Mus caroli*, which may be affected by the geographic and climatic differences between Mile City and Lianghe County. Mile City,^3^ located in the karst landform area of the Eastern Yunnan Plateau (103°04′–103°49′E, 23°50′–24°39′N), sits at an elevation of approximately 1,400 m. Its subtropical plateau monsoon climate features distinct dry and wet seasons, with annual precipitation ranging from 900 to 1,000 mm. Lianghe County,^4^ situated in the southern extension of the Hengduan Mountains’ river valleys in southwestern Yunnan (98°06′–98°31′E, 24°31′–24°58′N), spans elevations from 500 to 2,600 meters, creating vertical climate zones, characterized by a south subtropical monsoon climate, the county receives 1,300–1,600 mm of annual rainfall, maintains year-round humidity above 75%, and exhibits more pronounced contrasts between its extreme dry and wet seasons. Resulting in the differences in the distribution of rodent species.

The spleen tissue is usually used for the detection of *Bartonella*, and in this study, both spleen and kidney tissues were used for the detection, and a positive test was sufficient for one of the two. There was a statistically significant difference in the infection rate of *Bartonella* between the two tissues, with a higher rate of infection in the spleen than in the kidney, and only four strains of species were isolated from kidney tissues, whereas five were isolated from spleen tissues, which suggests that the spleen is more conducive for the growth and reproduction of *Bartonella*. This is inconsistent with the study results of Yu et al. (11) and Rao et al. (20), which showed no significant difference in the infection rate of various tissues. For the detection of infection in *Bartonella*, priority can be given to spleen tissue, and when conditions permit, multi-tissue testing is more conducive to accurately describing the true infection situation in rodents and enabling the acquisition of as many strain sequences as possible for analytical traceability.

In this study, the total infection rate of *Bartonella* in small mammals was 14.29%, which was similar to the rate of 14.9% (169/1,137) in southeastern China (21), higher than the rates of 7.9%–8.38% in Shandong Province (19, 22), 6.4% in Guangdong Province (10), and slightly lower than the rate of rodent *Bartonella* infection of 16.67% in Beitun area of Xinjiang (9), and significantly lower than 38.61% in the Qaidam Basin in western China (20) and 57.7% in Heilongjiang Province, China (7). The regional differences in *Bartonella* infection rates among small mammal species are the result of the combined effects of ecological characteristics at sampling sites, the selection of target organs for detection, and the methodological sensitivity employed. Current studies indicate that high-latitude regions (e.g., Heilongjiang and Beitun, Xinjiang) typically exhibit elevated *Bartonella* infection rates (7, 9, 20), potentially associated with arthropod vector activity and bacterial load in host organisms. Although Yunnan is situated at a lower latitude (24°N), it maintains a moderate infection level of 14.29% in this study area, likely attributable to its high rodent population density and exceptional species diversity (13), suggesting a compensatory effect of host community complexity on pathogen transmission. Regarding detection strategies, this study employed traditional PCR targeting the *gltA* gene in spleen and kidney tissues. Research from the Qaidam Basin demonstrated comparable infection rates between spleen/brain tissues (20), while studies in Beitun, Xinjiang revealed similar culture-positive rates in liver/spleen tissues (9). Collectively, although the spleen remains the preferred target organ for *Bartonella* detection (23), with significantly higher bacterial loads than other tissues (13), the low-load characteristics of kidney tissue may dilute overall detection rates. Furthermore, methodological differences substantially impact final detection outcomes: investigations in Heqing and Gongshan counties, Yunnan, utilizing *ssrA*-qPCR technology, achieved markedly higher sensitivity (31.5%) compared to conventional PCR (9.0%–1.4%) (13); Southeastern China studies enhanced detection reliability (14.9%) through dual-gene (*ssrA*/*gltA*) amplification (21); whereas research in Heilongjiang and the Qaidam Basin, combining bacterial culture with multi-gene sequencing (*gltA*, *ftsZ*, *rpoB*, etc.), proved more effective in capturing high-load or viable strains (7, 20).

Through comparative analysis of the geographical distribution of *Bartonella* in different regions, the research has confirmed that *Bartonella* infection is associated with the geographical distribution of host animals and is influenced by geographical environmental factors (7). The karst topography of Mile City, characterized by its caves, fissures, and hilly terrain, provides concealed habitats for rodents. In Lianghe County, the densely vegetated mountainous environment at the tropical margin, combined with persistently high humidity and an annual average temperature of around 20°C, sustains stable rodent populations. The dominant rodent species composition differs between these two regions, as does the *Bartonella* infection status in small mammals. Mile City captured 8 rodent species with 3 species testing positive for *Bartonella*, while Lianghe County captured 10 rodent species with 5 infected species. In addition, the prevalence of *Bartonella* among small mammals in Lianghe County in this study was 16.74% (39/233), although higher than 5.88% (4/68) in Mile City, and 3.79 folds higher than the infection risk in Mile City (95%*CI*: 1.39–13.35), it was significantly lower than 56.27% (229/407) shown by Luo et al. (8), which was found by comparison that there were as many as 18 species of small mammals infected with *Bartonella* in Lianghe County in their study, suggesting that increased rodent species richness may increase the risk of *Bartonella* transmitted by rodents.

Rodents are considered to be infected reservoir hosts for *Bartonella*, and differences in dominant rodent species in different regions may result in different local primary hosts for *Bartonella*. The prevalence of *Bartonella* in Inner Mongolia, China, was 47.27% (52/110), with *Eolagurus luteus* having the highest prevalence of 85.71% (18/21), followed by the *Spermophilus dauricus* at 47.62% (10/21), and the dominant species, *Meriones unguiculatus*, with a prevalence of 35.82% (24/67) (18). In this study, the highest infection rate was 75% (3/4) in *Niviventer confucianus*, followed by 33.33% (2/6) in *Rattus nitidus*; the dominant species of *Rattus tanezumi* was 22.07% (32/145), and the rate of *Suncus murinus* was 1.27% (1/79) and the risk of *Suncus murinus* infection was only 10% of that of the *Rattus tanezumi* (95% *CI*: 0.01–0.63), considering that the captured numbers of *Niviventer confucianus* and *Rattus nitidus* were small, they could not truly reflect the prevalence of *Bartonella* in the two species, and further studies were needed to verify it. The tail length of small mammals is also an influential factor in the prevalence of *Bartonella*, the longer the tail length, the greater the contact area provided to the pathogen, increasing the risk of infection. Tail length is one of the important external features for morphological identification of rodent species, and in particular, the tail length of adult rodents correlates with the species, confirming the influence of rodent species on the prevalence of *Bartonella*, which is consistent with the results of the *Rattus tanezumi* has a higher infection rate than that of the *Suncus murinus* and other rodent species.

Based on *gltA* gene sequencing, 57 strains were obtained from 43 murine animals, which were classified into five *Bartonella* species: *Bartonella tribocorum*, *Bartonella queenslandensis*, *Bartonella phoceensis*, *Bartonella mastomydis,* and *Bartonella clarridgeiae*. Among them, *Bartonella clarridgeiae* detected from *Rattus tanezumi* in Lianghe County is considered to be a zoonotic strain that may cause endocarditis (24). In addition, *Bartonella tribocorum* is genetically related to *Bartonella elizabethae*, which was isolated from endocarditis patients and identified as a zoonotic agent (25). It is suggested that residents of Lianghe County are at risk of infection with zoonotic bacterial strains of *Bartonella*, which warrants heightened vigilance. *Bartonella tribocorum* can be detected in *Rattus nitidus* and *Suncus murinus*, especially in *Rattus tanezumi*, so the main host of *Bartonella tribocorum* in this study was *Rattus tanezumi*. *Bartonella queenslandensis* strain has strong adaptability to different rodents and can be detected in *Rattus nitidus*, *Mus caroli*, *Rattus tanezumi*, *Niviventer confucianus,* and *Mus pahari*. *Bartonella phoceensis* was detected in *Rattus tanezumi* and *Rattus andamanensis*, in addition, *Bartonella mastomydis* and *Bartonella clarridgeiae* were detected only in *Rattus tanezumi*. Overall, five strains were isolated from *Rattus tanezumi*, suggesting that *Rattus tanezumi* are an important reservoir host for infection with *Bartonella* strains. The detection of multiple *Bartonella* in small mammals indicates that *Bartonella* has a high species diversity in small mammals. The same *Bartonella* can be detected in a variety of small mammals, indicating that *Bartonella* has a high adaptability in small mammals.

Co-infection (or mixed infection) refers to the simultaneous presence of at least two genetically distinct pathogens within the same host, this phenomenon not only reflects the coexistence of multiple infectious agents in hosts but also involves complex interactions between pathogens (26). In such infections, horizontal gene transfer and competitive inter-genotypic interactions among pathogens serve as critical evolutionary drivers for their survival strategies (27). The impact of mixed infections on host fitness is dual: while they may directly lead to atypical disease manifestations or increased host mortality, they can also indirectly regulate the transmission dynamics of pathogens by altering host susceptibility, infection probability, or transmission rates (26). Taking *Bartonella* as an example, co-infection systems may exhibit synergistic enhancement due to immune suppression, while ecological exclusion phenomena may arise from resource competition or cross-immunity (28). Previous studies have indicated potentially high co-infection rates in rodents (3). Our study revealed the concurrent detection of *Bartonella mastomydis* and *Bartonella tribocorum* in the same host species, the *Rattus tanezumi*, confirming the existence of *Bartonella* co-infections in small mammals. This finding aligns with a similar observation from the Qaidam Basin study, where two *Bartonella* species were isolated from different tissues of a *Meriones meridianus* (20). Further investigations into multi-tissue infections are warranted to better clarify the co-infection patterns of *Bartonella* in small mammals.

The limitations of this study are that the bacteria content in some tissue samples was small, the gel electrophoresis imaging showed positive initial results but failed to sequence, and the deviation of sequencing results reduced the number of representative strains. Additionally, the relatively limited sample size (a total of 301 rodents in Mile City and Lianghe County) may affect the accuracy of *Bartonella* infection rate assessment. Compared with similar studies in Yunnan Province, such as research in Jianchuan County, Yulong County, and Lianghe County based on 1,605 single-organ samples revealing an overall infection rate of 47.85% (8) and a study in Heqing County and Gongshan County with 333 samples achieving a 31.5% infection rate through multi-organ detection (13), the 14.29% overall infection rate observed here suggests insufficient sample size may weaken statistical power, potentially underestimating true infection levels or misjudging regional heterogeneity. Furthermore, the conventional PCR targeting the *gltA* gene used in this study exhibits technical limitations in sensitivity, struggling to identify low pathogen-load samples and non-*gltA*-dominant species, which may contribute to lower observed infection rates compared to high-prevalence regions. Future studies should expand the sampling scope, enhance sample representativeness, and implement multi-organ detection where feasible. To improve detection efficacy, a two-step strategy is recommended: initial high-efficiency screening using *Bartonella* genus-specific qPCR, followed by confirmatory testing of multiple conserved genes (e.g., *ssrA*, *rpoB*, *ftsZ*) via conventional or multiplex real-time PCR. Additionally, integrating molecular detection of multiple tissues and establishing a multi-locus sequence typing (MLST)-based analytical system will help systematically elucidate *Bartonella* epidemiology and genetic diversity in rodent populations. More sensitive and accurate sequencing methods should also be adopted to obtain stable results, aiding in characterizing antibacterial prevalence in murine animals.

The prevalence of *Bartonella* was higher among small mammals in Mile City and Lianghe County, Yunnan Province. Five species of *Bartonella* were isolated from the kidney or spleen tissues of 7 small mammals (*Rattus tanezumi*, *Suncus murinus*, *Niviventer confucianus*, *Rattus andamanensis*, *Mus pahari*, *Rattus nitidus,* and *Mus caroli*), including *Bartonella tribocorum*, *Bartonella queenslandensis*, *Bartonella phoceensis*, *Bartonella mastomydis* and *Bartonella clarridgeiae*, where *Bartonella tribocorum* is the dominant species in both regions. It is genetically related to *Bartonella elizabethae*, which was identified as a zoonotic pathogen early, and *Bartonella clarridgeiae* is also a zoonotic strain. The infection rate and isolated genotype of *Bartonella* in the spleen were higher than those in the kidney, which was more conducive to the growth and reproduction of *Bartonella*. The prevalence of *Bartonella* is affected by region, rodent species, and tail length. Therefore, it is very important to monitor the prevalence of *Bartonella* in rodents in different regions, and it is necessary to obtain a whole genome sequence as far as possible to identify *Bartonella* species more accurately, to better take targeted measures to prevent the infection of *Bartonella* transmitted by rodents to humans.

## Data Availability

The original contributions presented in the study are publicly available. These data can be found in GenBank at the National Center for Biotechnology Information (NCBI), accession numbers PQ279835-PQ279891.

## Footnotes

^1^ https://blast.ncbi.nlm.nih.gov/Blast.cgi

^2^ https://itol.embl.de/

^3^ https://www.hhml.gov.cn

^4^ https://www.dhlh.gov.cn/

## References

[1] Breitschwerdt EB. Bartonellosis, one health and all creatures great and small. Vet Dermatol. (2017) 28:96. doi: 10.1111/vde.12413, PMID: 28133871

[2] Gutiérrez R Krasnov B Morick D Gottlieb Y Khokhlova IS Harrus S. Bartonella infection in rodents and their flea ectoparasites: an overview. Vector Borne Zoonotic Dis. (2015) 15:27–39. doi: 10.1089/vbz.2014.1606, PMID: 25629778 PMC4307031

[3] Gutiérrez R Cohen C Flatau R Marcos-Hadad E Garrido M Halle S. Untangling the knots: co-infection and diversity of Bartonella from wild gerbils and their associated fleas. Mol Ecol. (2018) 27:4787–807. doi: 10.1111/mec.14906, PMID: 30357977

[4] Buhler KJ Fernando C Hill JE Galloway T Carriere S Fenton H. Combining deep sequencing and conventional molecular approaches reveals broad diversity and distribution of fleas and Bartonella in rodents and shrews from Arctic and subarctic ecosystems. Parasit Vectors. (2022) 15:366. doi: 10.1186/s13071-022-05446-w, PMID: 36229832 PMC9563109

[5] Demoncheaux J-P Medkour H Louni M Laugier L Pasqualini C Fenollar F. Detection of potential zoonotic Bartonella species in African Giant rats (*Cricetomys gambianus*) and fleas from an urban area in Senegal. Microorganisms. (2022) 10:489. doi: 10.3390/microorganisms10030489, PMID: 35336065 PMC8953472

[6] Mohd-Azami SNI Loong SK Khoo JJ Husin NA Lim FS Mahfodz NH. Molecular surveillance for vector-borne Bacteria in rodents and tree shrews of peninsular Malaysia oil palm plantations. Trop Med Infect Dis. (2023) 8:74. doi: 10.3390/tropicalmed8020074, PMID: 36828490 PMC9965954

[7] Li D-M Hou Y Song X-P Fu Y-Q Li G-C Li M. High prevalence and genetic heterogeneity of rodent-borne Bartonella species on Heixiazi Island, China. Appl Environ Microbiol. (2015) 81:7981–92. doi: 10.1128/AEM.02041-15, PMID: 26362983 PMC4651081

[8] Luo Y-Y Yu D Zhang H-Z Liu Z-X Hong R-D Hong M. Molecular detection of Bartonella species in wild small mammals in western Yunnan Province, China. Front Vet Sci. (2023) 10:1301316. doi: 10.3389/fvets.2023.1301316, PMID: 38076558 PMC10703294

[9] Xu A-L Chen Y-F Mu L Liu P-B Wang J Li R-X. Bartonella prevalence and genome sequences in rodents in some regions of Xinjiang, China. Appl Environ Microbiol. (2023) 89:e0196422. doi: 10.1128/aem.01964-22, PMID: 36951592 PMC10132096

[10] Yao X-Y Liu H Sun J Zhang Y-Q Lv Z-H Zhang X-L. Epidemiology and genetic diversity of Bartonella in rodents in urban areas of Guangzhou, southern China. Front Microbiol. (2022) 13:942587. doi: 10.3389/fmicb.2022.942587, PMID: 35859747 PMC9289675

[11] Yu J Xie B Bi G-Y Zuo H-H Du X-Y Bi L-F. Prevalence and diversity of small rodent-associated Bartonella species in Shangdang Basin, China. PLoS Negl Trop Dis. (2022) 16:e0010446. doi: 10.1371/journal.pntd.0010446, PMID: 35648747 PMC9159596

[12] Wang N Yin J-X Zhang Y Wu L Li W-H Luo Y-Y. Genetic evolution analysis and host characteristics of hantavirus in Yunnan Province, China. Int J Environ Res Public Health. (2022) 19:3433. doi: 10.3390/ijerph192013433, PMID: 36294012 PMC9603364

[13] Han P-Y Xu F-H Tian J-W Zhao J-Y Yang Z Kong W. Molecular prevalence, genetic diversity, and tissue tropism of Bartonella species in small mammals from Yunnan Province, China. Animals. (2024) 14:1320. doi: 10.3390/ani14091320, PMID: 38731324 PMC11083988

[14] Ji X Wang M Li L Chen F Zhang Y Li Q. The impact of repeated freeze-thaw cycles on the quality of biomolecules in four different tissues. Biopreserv Biobanking. (2017) 15:475–83. doi: 10.1089/bio.2017.0064, PMID: 28930488

[15] Norman AF Regnery R Jameson P Greene C Krause DC. Differentiation of Bartonella-like isolates at the species level by PCR-restriction fragment length polymorphism in the citrate synthase gene. J Clin Microbiol. (1995) 33:1797–803. doi: 10.1128/jcm.33.7.1797-1803.1995, PMID: 7545181 PMC228273

[16] Malania L Bai Y Osikowicz LM Tsertsvadze N Katsitadze G Imnadze P. Prevalence and diversity of Bartonella species in rodents from Georgia (Caucasus). Am J Trop Med Hygiene. (2016) 95:466–71. doi: 10.4269/ajtmh.16-0041, PMID: 27162268 PMC4973202

[17] Jian R Ren Q Xue J Xie G-C Wang J Chen G-Q. Genetic diversity of Bartonella infection in residential and field rodents in Hebei, China. Front Microbiol. (2022) 13:1039665. doi: 10.3389/fmicb.2022.1039665, PMID: 36504836 PMC9732461

[18] Li J Zhang C Lu M Wang Y Wang W Liu F. The diverse genetic genotypes of Bartonella species circulating in rodents from Inner Mongolia, northern China. PLoS Negl Trop Dis. (2023) 17:e0011462. doi: 10.1371/journal.pntd.0011462, PMID: 37384796 PMC10337887

[19] Qin X-R Liu J-W Yu H Yu X-J. Bartonella species detected in rodents from eastern China. Vector Borne Zoonotic Dis. (2019) 19:810–4. doi: 10.1089/vbz.2018.2410 31355717

[20] Rao H Li S Lu L Wang R Song X Sun K. Genetic diversity of Bartonella species in small mammals in the Qaidam Basin, western China. Sci Rep. (2021) 11:1735. doi: 10.1038/s41598-021-81508-w, PMID: 33462399 PMC7814127

[21] Liu H Han T Liu W Xu G Zheng K Xiao F. Epidemiological characteristics and genetic diversity of Bartonella species in rodents from southeastern China. Zoonoses Public Health. (2022) 69:224–34. doi: 10.1111/zph.12912, PMID: 35040279

[22] Zhang L Peng Q Gu X-L Su W-Q Cao X-Q Zhou C-M. Host specificity and genetic diversity of Bartonella in rodents and shrews from eastern China. Transbound Emerg Dis. (2022) 69:3906–16. doi: 10.1111/tbed.14761, PMID: 36355627

[23] Gutiérrez R Vayssier-Taussat M Buffet J-P Harrus S. Guidelines for the isolation, molecular detection, and characterization of Bartonella species. Vector Borne Zoonotic Dis. (2017) 17:42–50. doi: 10.1089/vbz.2016.1956 28055575

[24] Eremeeva ME Gerns HL Lydy SL Goo JS Ryan ET Mathew SS. Bacteremia, fever, and splenomegaly caused by a newly recognized bartonella species. N Engl J Med. (2007) 356:2381–7. doi: 10.1056/NEJMoa065987, PMID: 17554119

[25] Heller R Riegel P Hansmann Y Delacour G Bermond D Dehio C. *Bartonella tribocorum* sp. nov., a new Bartonella species isolated from the blood of wild rats. Int J Syst Bacteriol. (1998) 48:1333–9. PMID: 9828434 10.1099/00207713-48-4-1333

[26] Hoarau AOG Mavingui P Lebarbenchon C. Coinfections in wildlife: focus on a neglected aspect of infectious disease epidemiology. PLoS Pathog. (2020) 16:e1008790. doi: 10.1371/journal.ppat.1008790, PMID: 32881983 PMC7470396

[27] Abbot P Aviles AE Eller L Durden LA. Mixed infections, cryptic diversity, and vector-borne pathogens: evidence from Polygenis fleas and Bartonella species. Appl Environ Microbiol. (2007) 73:6045–52. doi: 10.1128/AEM.00228-07, PMID: 17693558 PMC2075021

[28] Telfer S Lambin X Birtles R Beldomenico P Burthe S Paterson S. Species interactions in a parasite community drive infection risk in a wildlife population. Science. (2010) 330:243–6. doi: 10.1126/science.1190333, PMID: 20929776 PMC3033556

